# Bronchoscopic Diagnosis and Treatment of Primary Tracheobronchial Amyloidosis: A Retrospective Analysis from China

**DOI:** 10.1155/2017/3425812

**Published:** 2017-01-19

**Authors:** Xiaoxiao Lu, Bixiu He, Ge Wang, Baimei He, Lijing Wang, Qiong Chen

**Affiliations:** ^1^Department of Geriatrics, Respiratory Medicine, Xiangya Hospital, Central South University, Changsha, Hunan 410008, China; ^2^Department of Information, Zhengzhou Maternal and Children Health Hospital, Zhengzhou, Henan 450012, China

## Abstract

*Objective*. To assess the value of bronchoscopy in the diagnosis and treatment of primary tracheobronchial amyloidosis (TBA), in order to reduce misdiagnosis rates and improve prognosis.* Methods*. Clinical data of 107 patients with TBA reported from 1981 to 2015 in China were retrospectively analyzed for clinical features, bronchoscopic manifestations, pathologies, treatments, and outcomes.* Results*. 105 of 107 TBA patients were pathologically confirmed by bronchoscopy. Main bronchoscopic manifestations of TBA were single or multiple nodules and masses within tracheobronchial lumens; local or diffuse luminal stenosis and obstruction; luminal wall thickening and rigidity; rough or uneven inner luminal walls; congestion and edema of mucosa, which was friable and prone to bleeding upon touch; and so forth. 53 patients were treated with bronchoscopic interventions, like Nd-YAG laser, high-frequency electrotome cautery, freezing, resection, clamping, argon plasma coagulation (APC), microwaving, stent implantation, drug spraying, and other treatments. 51 patients improved, 1 patient worsened, and 1 died.* Conclusion*. Bronchoscopic biopsy is the primary means of diagnosing TBA. A variety of bronchoscopic interventions have good short-term effects on TBA. Bronchoscopy has important value in the diagnosis, severity assessment, treatment, efficacy evaluation, and prognosis of TBA.

## 1. Introduction

Primary tracheobronchial amyloidosis (TBA) refers to a group of rare diseases with varied clinical manifestations which are caused by abnormal deposition of *β*-sheet amyloids in the tracheal and bronchial submucosa in the absence of systemic amyloidosis. Incidence of amyloidosis is about 8/1 million [1]. TBA accounts for 1.1% of all amyloidoses [2]. In China, only over 200 cases of TBA have been reported so far. The disease lacks typical clinical symptoms and imaging manifestations [3], so if clinicians do not have sufficient knowledge about TBA, misdiagnosis and missed diagnosis will be highly likely [4]. In recent years, with the popularization of bronchoscopy, the number of TBA cases diagnosed by bronchoscopy has significantly increased than before. The rapid development of bronchoscopic interventional techniques has provided TBA patients with new treatment options. This paper aims to retrospectively analyze 2 cases of TBA patients in our hospital in recent years as well as 107 TBA patients reported from 1981 to 2015 in China, with the focus on explaining the value of bronchoscopy in the diagnosis and treatment of TBA.

## 2. Subjects and Methods

### 2.1. Retrieval Methods

With “primary tracheobronchial amyloidosis” as search term, CNKI, VIP, and Wanfang Databases were retrieved. Meanwhile, PubMed database was searched with “primary tracheobronchial amyloidosis” and “China” as search terms. Data collected included age, gender, disease duration, clinical manifestations, presence or absence of misdiagnosis, imaging manifestations, bronchoscopic manifestations, pathology, treatments, and prognosis.

### 2.2. Screening Methods

Inclusion criteria were (1) patients histopathologically diagnosed with TBA and (2) those with complete clinical data. Exclusion criteria were (1) duplicate reports; (2) secondary tracheobronchial amyloidosis or systemic amyloidosis; and (3) patients with accompanying pharyngolaryngeal or pulmonary parenchymal amyloidosis. According to the inclusion and exclusion criteria, we screened out 54 papers [5–28] from the four databases to enroll 107 TBA patients, which totaled 109 cases after adding the 2 cases in our hospital.

### 2.3. Data Analysis Methods

Data were analyzed using SPSS 17.0 statistical software. Normally distributed measurement data were expressed as mean ± SD; abnormally distributed measurement data were expressed as median, while enumeration data were presented as constituent ratio.

## 3. Case Information

### 3.1. Case 1

The patient is a 68-year-old male who complained of recurrent cough, expectoration, and progressive dyspnea for more than 30 years. He was admitted to our hospital after being diagnosed with chronic bronchitis, bronchiectasis, and endobronchial tuberculosis in other hospitals and treated with antibiotics numerous times. Despite treatment, his symptoms progressively worsened. In 1988, the patient was managed with a combination of isoniazid, streptomycin, and rifampicin for a diagnosis of endobronchial tuberculosis after bronchoscopy in another hospital. Three months later, with no improvement on these antitubercular agents and seeking a second opinion and treatment, the patient discontinued the treatment and came to our hospital.

**Figure 1:**
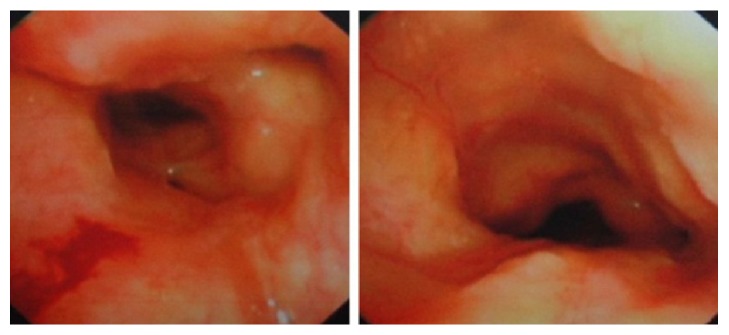
Electronic bronchoscopy photographs of the first patient. Pictures above reveal significant swelling and hypertrophy of the tracheal and bronchial mucosa.

The patient presented with diminished breath sounds and slight bibasilar wheezing rales and crackles. Routine blood test was normal. A PPD test was negative. Sputum samples were negative for bacteria, acid-fast bacilli, and fungi. A recent chest CT demonstrated extensive thickening of the walls of the trachea and bronchi at different levels with luminal narrowing. Electronic bronchoscopy revealed significant swelling and hypertrophy of the tracheal and bronchial mucosa (Figure 1). Pathological examination of the endobronchial biopsy specimen revealed chronic inflammation of the mucosa with amyloid deposition, PAS stain (−), digestive PAS stain (−), and Congo red stain (+). There was no evidence of extrapulmonary organ involvement in amyloidosis. Based on the above comprehensive evaluation, the diagnosis of primary TBA was established.

### 3.2. Case 2

**Figure 2:**
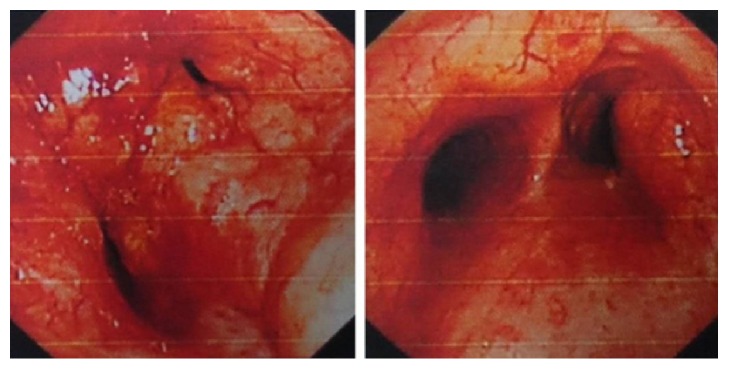
Electronic bronchoscopy photographs of the second patient. Pictures above reveal mucosal hypertrophy, swelling, significant hyperemia, unevenness and luminal stenosis in the trachea and bronchi, and total occlusion of right middle lobe bronchus.

A 53-year-old male patient was admitted because of recurrent cough, expectoration, bloody sputum for 20 years, and shortness of breath for 5 years. He had been repeatedly diagnosed with bronchiectasis and chronic bronchitis in other hospitals. Physical examination on admission revealed moist rales in bilateral lower lungs. Routine blood test was normal. Tuberculosis antibody was negative. *G* test was negative. Chest CT showed thickening and calcification of main bronchial wall as well as left and right bronchial walls and secondary atelectasis of right middle lobe. Electronic bronchoscopy findings were mucosal hypertrophy, swelling, significant hyperemia, unevenness and luminal stenosis which were visible in the trachea, left and right main bronchi, right upper lobe and right intermediate bronchi, right lower lobe bronchus, as well as left upper and lower lobe bronchi, and total occlusion of right middle lobe bronchus (Figure 2). Pathological examination results showed chronic mucosal inflammation and amyloid deposition, acid-fast staining (−), digested PAS staining (−), and Congo red staining (+). No evidence of amyloidosis was observed in other parts. The patient was diagnosed with primary TBA, who was treated with melphalan 10 mg Qd, methylprednisolone 40 mg Qd, and bronchoscopic Nd-YAG laser twice to get markedly relieved symptoms. His condition was stable during a 6-month follow-up.

## 4. Results 

### 4.1. General Information

Among 109 patients included, 23 cases were reported before the year 2000, while 86 cases were reported after 2000. These patients consisted of 67 males and 42 females, with a male/female ratio = 1.6 : 1. Age of onset ranged between 18 and 81 years, with a mean of (52.16 ± 11.33) years. As for disease duration, the longest was 30 years, whereas the shortest was 12 hours, with a median of 2 years. Main clinical manifestations were cough, shortness of breath or progressive dyspnea, expectoration, hemoptysis or bloody sputum, fever, chest tightness, hoarseness, and so forth. Misdiagnosis rate at first hospitalization was up to 45%. Most patients were misdiagnosed as chronic bronchitis, endobronchial tuberculosis, lung tumor, bronchial asthma, pulmonary infections, and so forth (see Table 1 for details).

### 4.2. Imaging Manifestations

82 patients underwent lung CT examination, who were manifested mainly with tracheal stenosis, tracheal wall thickening, calcification of wall, patchy shadows, atelectasis, and hilar space-occupying lesions. 59 patients underwent chest X-ray examination, who were manifested often with increased and disorderly lung markings, atelectasis, patchy shadows, bronchitis, emphysema, luminal stenosis, and enlarged hilar shadows (see Table 2 for details).

### 4.3. Bronchoscopic and Histopathological Examinations

107 patients underwent bronchoscopy. Main manifestations included multiple nodules or masses within tracheal and bronchial lumens, luminal stenosis or occlusion, bronchial wall thickening, brittle and easily bleeding mucosa, mucosal roughness or unevenness, mucosal hyperemia and edema, pale mucosa, and wall rigidity. Histopathology of all patients showed amyloid deposition in tracheal and bronchial submucosa. Among them, brick red amyloid was reported in 50 cases by Congo red staining, while only 22 cases mentioned observation of yellow-green birefringence of amyloid under polarizing microscope (see Table 3 for details).

### 4.4. Treatments and Outcomes

53 patients were treated with bronchoscopic interventions, including Nd-YAG laser, argon plasma coagulation (APC), freezing, drug spraying, clamping, resection, high-frequency electrotome cautery, stent implantation, and microwaving. Among these patients, 20 received bronchoscopic therapy alone; 32 received bronchoscopic therapy combined with drug therapy; while 1 received bronchoscopic therapy combined with external beam radiation therapy (EBRT). 51 patients improved, 1 patient worsened, and 1 died. Meanwhile, 25 patients received drug therapy alone. Among them, 20 patients improved, 4 patients worsened, and 1 died. 2 patients were given EBRT, both of whom improved. 2 patients underwent surgical resection and improved. 27 patients were given symptomatic treatment or only clinical observation, of whom 5 aggravated and 2 died. 66 patients were followed up for more than 3 months. Improvement was found in 2 patients; stabilization was found in 47 patients; exacerbation was found in 9 patients; and death was found in 8 patients (commonly died of lung infection, massive hemorrhage, and respiratory failure) (see Table 4 for details).

## 5. Discussion

Amyloidosis is a group of clinical syndromes caused by deposition of insoluble amyloid fibrils in extracellular matrix, which produces mass effect and cytotoxicity, thereby damaging normal tissue and organ structures and ultimately leading to target organ dysfunction [29]. Amyloidosis may affect various tissues and organs, commonly kidneys, heart, liver, nervous system, and skin, while very rarely it affects trachea and bronchi.

In 1877, Lesser first reported respiratory amyloidosis [30], but so far its specific pathogenesis is unclear, which has been speculated by some scholars to be associated with immune-related disorders such as plasma cell clone and excessive immunoglobulin secretion [31–33]. TBA is a latent chronic disease. We found through retrospective analysis that the duration of TBA can exceed 30 years and that up to 25% of patients had TBA duration longer than 5 years. Our data show that the disease is prevalent among elderly people, with a peak onset age of 50–60 years, which is slightly more common in men than in women as well [34]. Clinical manifestations of TBA patients are closely related to the sites and severity of lesions. Common symptoms include cough, shortness of breath, progressive dyspnea, expectoration, and hemoptysis. TBA mainly affects the trachea, main bronchus, lobar bronchi, and segmental bronchi, while it has little impact on small airways and alveoli. Thus, TBA is generally manifested as varying degrees of obstructive ventilatory dysfunction by lung function test. Chest X-ray manifestations are generally increased, disorderly, or normal lung markings or patchy shadows and atelectasis when accompanied with obstructive pneumonia. Chest CT has certain advantages compared to chest X-ray [35], but for early amyloidosis with no obvious bronchial thickening or stenosis, its diagnostic effect is not very satisfactory. Soussan et al. has reported that FDG PET/CT might be valuable for the evaluation of tracheobronchial amyloidosis metabolic activity and follow-up [36]. However, it has not been promoted due to the high expenses. As clinical manifestations and imaging features of TBA lack specificity, it is often misdiagnosed as chronic bronchitis, endobronchial tuberculosis, lung tumor, bronchial asthma, bronchiectasis, and so forth. Statistics of data in this paper show that initial misdiagnosis rate of TBA is up to 45%, and for the two patients diagnosed in our hospital, one was misdiagnosed for 20 years, whereas the other was misdiagnosed for as long as 30 years.

Introduction and extensive application of bronchoscopy have shed a new light on the diagnosis and treatment of TBA. Through literature review, we found that among TBA cases included in this study, only 23 were reported before 2000, while the number of cases reported since 2000 increased to 86 cases. For this phenomenon, we believe that improved TBA diagnostic technique is a factor that cannot be ignored aside from clinicians' increased awareness of the disease, especially the development and popularization of bronchoscopic techniques in the past decade, which play a vital role [37]. Bronchoscopy not only allows comprehensive and intuitive observation of the sites, types, involving scope and severity of tracheal and bronchial lesions, but also enables condition assessment according to the extent of disease, degree of airway stenosis, and presence or absence of complications such as obstructive pneumonia and airway hemorrhage in patients, based on which prognosis is evaluated. According to a literature report [38], patients with lesions involving the proximal airway and severe middle airway involvement had poor outcomes, most of whom died of progressive aggravated respiratory failure, pulmonary infection, and massive hemorrhage. Depending on the scope of tracheobronchial amyloid deposition and characteristics of lesions, bronchoscopic manifestations of TBA can be classified into three types: (1) multifocal tracheal, bronchial submucosal plaques (the most common type); (2) unifocal nodule or tumor-like mass; and (3) diffuse or infiltrative [39]. TBA has distinct morphological features under bronchoscopy, which are easier to identify. Common manifestations include single or multiple nodules and masses within tracheal and bronchial lumens, localized or diffuse luminal stenosis and occlusion, wall thickening and rigidity, rough or uneven inner luminal wall, mucosal hyperemia and edema, and brittle and easily bleeding mucosa. Narrow-band imaging (NBI) bronchoscopy launched in recent years is an emerging bronchoscopic technique, which is mainly used for the diagnosis of early central lung cancer and precancerous lesions. Serrano-Fernández et al. [40] found that if three vascular signs, capillary loops, formation of complex vascular networks, and sudden interruption of large blood vessels, were observed under NBI bronchoscopy, TBA should be highly possible, which could be differentiated from bronchopulmonary cancer as well. Since NBI bronchoscopy is rather sensitive to early TBA and can detect minute lesions, it is conducive to early diagnosis and intervention and helps improve patient prognosis.

Histopathological biopsy is the gold standard for diagnosis of TBA, while bronchoscopic biopsy is the primary means of confirming TBA diagnosis. Compared to the open lung biopsy, bronchoscopic biopsy has advantages such as small invasion, easy and accurate sampling, and little complications. 107 of 109 patients analyzed in this study were diagnosed by bronchoscopic biopsy, which sufficiently shows the significance of bronchoscopy in diagnosing TBA. Histologically, amyloids under tracheobronchial mucosa were presented as homogeneous, amorphous eosinophilic substance by HE staining, which showed brick red color under light microscope by Congo red staining and yellow-green birefringence under polarizing microscope [41].

At present, major treatments of TBA are drug therapy, surgical resection, EBRT [42], and bronchoscopic interventions, as well as temporary follow-up observation (for asymptomatic patients). Since the mid-1990s, the Boston University Amyloidosis Center has used myeloablative-dose melphalan plus autologous hematopoietic stem-cell transplantation; however, up to 15% of patients experienced severe complications [43, 44]. Despite good efficacy of classical chemotherapy melphalan-prednisone (MP regimen) for systemic amyloidosis, its efficacy for primary TBA is uncertain [45]. Surgical resection is only suitable for TBA patients in general good condition with localized lesions but has the risk of hemorrhage [46]. Bronchoscopic interventions for treating TBA include Nd-YAG laser [47], high-frequency electrotome cautery, freezing, resection, clamping, APC, microwaving, stent implantation [48], and drug spraying [37], which can be used in combination with other treatments. In the literatures statistically analyzed herein, most of patients who received bronchoscopic interventions got the desired short-term effect [49]. However, data for evaluating long-term efficacy are still lacking, so regular bronchoscopic reexamination can be performed to evaluate the therapeutic effect. Although bronchoscopic interventions can quickly relieve the symptoms, multiple repeated interventions are often required as TBA can relapse.

In summary, when we encounter unexplained chronic cough, progressive dyspnea, and recurrent noninfectious obstructive pneumonia in clinical practice, the possibility of TBA should be considered [50]. This will remind us to further confirm the diagnosis by bronchoscopy or by biopsy, if necessary. Bronchoscopy has important value in the diagnosis, condition assessment, treatment, and efficacy evaluation of TBA.

## Figures and Tables

**Table 1:** General information of patients.

Feature	*N* = 109	%
Report time		
Years 1981–1999	23/109	21.1
Years 2000–2015	86/109	78.9
Gender		
Male	67/109	61.5
Female	42/109	38.5
Age of onset		
≤65 years	16/109	14.7
>65 years	93/109	85.3
Disease duration (*n* = 60)		
≤5 years	45/60	75.0
>5 years	15/60	25.0
Clinical manifestations		
Cough	76/109	69.7
Shortness of breath	67/109	61.5
Expectoration	46/109	42.2
Hemoptysis	33/109	30.3
Fever	17/109	15.6
Chest tightness	10/109	9.2
Hoarseness	7/109	6.4
Chest pain	2/109	1.8
Fatigue	2/109	1.8
Misdiagnosis at first hospitalization	49/109	45.0
Chronic bronchitis	18/109	16.5
Endobronchial tuberculosis	8/109	7.3
Lung tumor	7/109	6.4
Bronchial asthma	7/109	6.4
Pulmonary infection	6/109	5.5
COPD	2/109	1.8
Bronchiectasis	1/109	0.9

COPD: chronic obstructive pulmonary disease.

**Table 2:** Imaging manifestations.

Feature	*N* = 109	%
Lung CT (*n* = 82)		
Tracheal stenosis	47/82	57.3
Tracheal wall thickening	42/82	51.2
Wall calcification	17/82	20.7
Patchy shadows	15/82	18.3
Atelectasis	13/82	15.9
Hilar space-occupying lesions	6/82	7.3
Normal	11/82	13.4
Chest X-ray (*n* = 59)		
Increased, disorderly lung markings	13/59	22.0
Atelectasis	11/59	18.6
Patchy shadows	8/59	13.6
Bronchitis	5/59	8.5
Emphysema	5/59	8.5
Luminal stenosis	5/59	8.5
Enlarged hilar shadows	4/59	6.8
Normal	16/59	27.0

CT: computerized tomography.

**Table 3:** Bronchoscopic and histopathological examinations.

Feature	*N* = 109	%
Bronchoscopy (*n* = 107)		
Multiple intraluminal nodules, masses	64/107	59.9
Luminal stenosis, occlusion	54/107	50.5
Bronchial wall thickening	30/107	28.0
Brittle and easily bleeding mucosa	26/107	24.3
Mucosal unevenness	23/107	21.5
Mucosal hyperemia and edema	22/107	20.6
Mucosal paleness	1/107	0.9
Wall rigidity	1/107	0.9
Histopathology (*n* = 109)		
Submucosal amyloid deposition	109/109	100.0
Brick red amyloid by Congo red staining	50/109	45.9
Yellow-green birefringence of amyloid by polarizing microscopy	22/109	20.2

**Table 4:** Treatments and outcomes.

Feature	*N* = 109	Outcomes		
		Improvement	Exacerbation	Death
Means of bronchoscopic therapy (*n* = 53)		51	1	1
Nd-YAG laser	19/53			
APC	18/53			
Freezing	15/53			
Drug spraying	12/53			
Clamping	6/53			
Resection	5/53			
High-frequency electrotome cautery	4/53			
Stent implantation	2/53			
Microwaving	1/53			
Bronchoscopy/other treatments (*n* = 53)		51	1	1
Bronchoscopic therapy alone	20/53			
Combined with drug therapy	32/53			
Combined with EBRT	1/53			
Drug therapy alone (*n* = 25)	25/25	20	4	1
EBRT/other treatments (*n* = 2)		2	0	0
Combined with glucocorticoids	1/2			
Combined with glucocorticoids + colchicine	1/2			
Surgical resection (*n* = 2)	2/2	2	0	0
Clinical observation/symptomatic treatment (*n* = 27)	27/27	20	5	2
Follow-up ≥ 3 months	66/109	49	9	8

APC: argon plasma coagulation; EBRT: external beam radiation therapy.
